# Bioactive Compounds of Food: Their Role in the Prevention and Treatment of Diseases

**DOI:** 10.1155/2019/3765986

**Published:** 2019-03-11

**Authors:** Anderson Junger Teodoro

**Affiliations:** Universidade Federal do Estado do Rio de Janeiro (UNIRIO), Laboratory of Functional Foods, Rio de Janeiro, Brazil

The aim of this special issue on “Bioactive Compounds of Food: Their Role in the Prevention and Treatment of Diseases” is to provide a representation of the new trends of bioactive compounds of food involved in different pathologies. Food bioactive compounds are extranutritional constituents that typically occur in small quantities in foods. In summary, numerous bioactive compounds appear to have beneficial health effects. Much scientific research needs to be conducted before we can begin to make science-based dietary recommendations. Despite this, there is sufficient evidence to recommend consuming food sources rich in bioactive compounds. From a practical perspective, this translates to recommending a diet rich in a variety of fruits, vegetables, whole grains, legumes, oils, and nuts. Therefore, the current issue will be focused on identifying as well as understanding the mechanistic role of food bioactive compounds in a range of human pathologies. This issue is logically divided into four main areas: (a) bioactive compounds and inflammation, (b) functional food in chronic diseases, (c) bone health and polyphenols, and (d) neuroprotective effects of bioactive compounds. Forty-four papers were selected through our routine rigorous double-blind external peer review by qualified experts. Due to the capacity limitation, some papers have already appeared earlier in the last issue (volume 2019, No. 2019). Some papers are briefly introduced below.

## 1. Bioactive Compounds and Inflammation

Inflammation is the major cause and aggravating factor of various acute or chronic pathological conditions, including photoaging, diabetes, and cancer. The inflammatory response promotes the activation of transcriptional factors and proinflammatory cytokines, which can lead to an unresolved inflammatory response associated with an inhibition of insulin signaling and high risk for cardiovascular events. Epidemiological and intervention studies have been carried out to find out dietary patterns, foods, and bioactive compounds with protective anti-inflammatory actions. Thus, a way to prevent inflammation which can lead to carcinogenesis or cardiovascular diseases is through the use of bioactive food compounds of spices and herbs which show both antioxidant and anti-inflammatory properties. For this reason, anti-inflammatory phytochemicals could represent an exogenous aid crucial for the prevention of chronic diseases mediated by inflammatory processes.

An important paper by A. Kicel et al. (published in Vol. 2018) entitled “Multifunctional Phytocompounds in *Cotoneaster* Fruits: Phytochemical Profiling, Cellular Safety, Anti-Inflammatory and Antioxidant Effects in Chemical and Human Plasma Models *In Vitro*” provide a more detailed insight into the chemical composition and activity of *Cotoneaster* fruits. To this end, the fruits from nine species of *Cotoneaster* cultivated in Poland were analyzed for a range of lipophilic and hydrophilic (polyphenolic) constituents with acknowledged health-promoting properties using a combination of chromatographic and spectroscopic methods (GC-FID-MS, UHPLC-PDA-ESI-MS3, and UV-Vis spectrophotometry). The most promising polyphenolic fractions were then subjected to an analysis of antioxidant activity comprising eight complementary in vitro tests (both chemical and biological plasma models) covering some of the mechanisms crucial for reducing the level of oxidative damage in the human organism, that is, scavenging of free radicals, enhancement of the nonenzymatic antioxidant capacity of blood plasma, and protection of its lipid and protein components against oxidative/nitrative changes. Additionally, the inhibitory effects of the fruit extract on the proinflammatory enzymes, that is, lipoxygenase and hyaluronidase, were also measured. Hence, *Cotoneaster* fruits appear to be promising candidates for the production of pharma- and nutraceuticals associated with preventing and treating oxidative stress and inflammatory-related chronic diseases; they may also contribute to a balanced and varied diet comprising food rich in bioactive compounds.

Falcarinol (FA) and falcarindiol (FD) are the most abundant carrot-derived polyacetylenes and have a demonstrated anti-inflammatory effect, in part by the suppression of NF*κ*B. FD has been shown to activate Nrf2 by S-alkylation of its inhibitor protein Keap1. A. Stefanson and M. Bakovic (published in Vol. 2018) evaluate for the first time the protective effect of diet-achievable levels of FA against intestinal inflammation in comparison to sulforaphane (SF)—widely recognized as the most potent natural compound activator of the Nrf2/ARE pathway. The results are described in the paper “Falcarinol Is a Potent Inducer of Heme Oxygenase-1 and Was More Effective than Sulforaphane in Attenuating Intestinal Inflammation at Diet-Achievable Doses.”

Sepsis is an organ dysfunction that results from a dysregulated host response to infection. It is known that there is an interrelation between redox processes and inflammation and that reactive species can activate inflammatory signaling pathways and that inflammatory cells can produce more reactive species, resulting in a vicious cycle leading to a redox and inflammatory disequilibrium, and thus may be a determining factor in the sepsis outcome. Thus, the interaction between redox processes and inflammation would be a determining factor for the antioxidant selection with therapeutic potential to minimize systemic damage and improve the septic animal's survival. K. P. Lúcio et al. (published in Vol. 2018) evaluate the anti-inflammatory and antioxidant properties of *Morus nigra* L. in a sepsis model induced by LPS. Male C57BL/6 mice were distributed into four groups: control, sepsis, sepsis treated with leaf extract of mulberry, and sepsis treated with mulberry pulp. The animals were treated with 100 *μ*L of their respective treatments for twenty-one days. Sepsis was induced at the 21st day with lipopolysaccharide (LPS) by intraperitoneal injection. The results of the paper “Anti-Inflammatory and Antioxidant Properties of Black Mulberry (*Morus nigra* L.) in a Model of LPS-Induced Sepsis” showed that the treatment with the extracts of leaves and the pulp of *Morus nigra* produced beneficial effects on the modulation of important parameters that are normally altered in sepsis.

Different strategies for the treatment of sepsis have emerged in the last few years, but none of them has proven to be beneficial in clinical trials. Lipids can modulate leukocyte function and therefore the immune response. Omega-9 is a natural agonist of peroxisome proliferator-activated receptor (PPAR). PPAR gamma ligands had been demonstrated to protect septic animals against microvascular dysfunction and enhance bacterial elimination through neutrophil extracellular trap formation. The paper entitled “Omega-9 Oleic Acid, the Main Compound of Olive Oil, Mitigates Inflammation during Experimental Sepsis” by I. M. Medeiros-de-Moraes et al. (published in Vol. 2018) investigated the effect of Omega-9 on systemic corticosterone levels, inflammatory markers, cell migration, bacterial clearance, and nuclear receptor PPAR gamma expression in both liver and adipose tissues during experimental sepsis. The authors also studied Omega-9 effects on leukocyte rolling in vivo. It has been demonstrated that Omega-9 modulated the immune response in septic mice. Omega-9 decreased the production of proinflammatory cytokines, increased IL-10 production, reduced neutrophil migration and accumulation in the site of infection, and also improved bacterial clearance. Omega-9 treatment affected leukocyte trafficking in septic animals and inflamed cremaster muscle postcapillary venules by decreasing selectin-dependent leukocyte rolling in vivo.

## 2. Functional Food in Chronic Diseases

Obesity, insulin resistance, hypertension, chronic inflammation, dyslipidemia, and oxidative stress are considered the major risk factors for different pathologies. The paper entitled “Gamma Oryzanol Treats Obesity-Induced Kidney Injuries by Modulating the Adiponectin Receptor 2/PPAR-*α* Axis” evaluated the effect of *γ*Oz to recover renal function in obese animals by high sugar-fat diet by the modulation of the adiponectin receptor 2/PPAR-*α* axis. F. V. Francisqueti et al. (published in Vol. 2018) concluded that Oz is able to modulate PPAR-*α* expression, inflammation, and oxidative stress pathways improving obesity-induced renal disease.

Hypertension is a dominant risk factor for chronic diseases, including cardiovascular disorders, stroke, renal diseases, and diabetes. Hypertension is the second leading cause of disability around the world. Bioactive phytoconstituents, available as natural components in foods and medicinal plants, provide preventive and curative health benefits to improve cardiovascular health. The functionalities of bioactives from green resources, including the inhibition of the activity of enzymes or the formation of complexes with metals, which catalyze the oxidation reaction and the capacity to modulate metabolic processes, may result in the eradication and management of cardiovascular diseases. F. Hussain et al. (published in Vol. 2018) investigated these themes by identifying and characterizing the bioactive compounds of *Coriandrum sativum* responsible for the treatment of hypertension through LC-ESI-MS/MS and by exploring their mechanism of action as angiotensin-converting enzyme (ACE) inhibitors in the work “Identification of Hypotensive Biofunctional Compounds of *Coriandrum sativum* and Evaluation of Their Angiotensin-Converting Enzyme (ACE) Inhibition Potential.”

The incidence of diabetes mellitus (DM), a metabolic disturbance disease characterized by chronic hyperglycemia, has increased rapidly worldwide. Currently, a global population of 382 million people is diagnosed with DM and this number is predicted to rise to 592 million by 2035. Current antidiabetic therapies have some limitations. Moreover, DM is a chronic disease with miscellaneous complications that require long-term treatment. Some effective Western medicines for diabetes are associated with high cost and adverse effects. Therefore, it is necessary to find alternative agents for the treatment of diabetes and its complications that have lower costs and fewer side effects. Phytochemicals are regarded as an important source for treating human health problems, including DM. Foods are composed of a variety of bioactive substances such as polysaccharides, pigments, minerals, peptides, and polyphenols, which have valuable pharmaceutical and biomedical potential. In these context, the papers “*Polysiphonia japonica* Extract Attenuates Palmitate-Induced Toxicity and Enhances Insulin Secretion in Pancreatic Beta-Cells”(S.-H. Cha et al., published in Vol. 2018), “The Antidiabetic and Antinephritic Activities of *Tuber melanosporum* via Modulation of Nrf2-Mediated Oxidative Stress in the db/db Mouse” (X. Jiang et al., published in Vol. 2018), and “Inhibitory Effects of Momordicine I on High-Glucose-Induced Cell Proliferation and Collagen Synthesis in Rat Cardiac Fibroblasts” (P.-Y. Chen et al., published in Vol. 2018) explored the antidiabetic properties of bioactive compounds with different matrices and found that the effects may be related to the modulation of oxidative stress and inflammation-related cytokines via Nrf2 signaling and may improve insulin secretion. The effects may provide these compounds with a candidacy for a natural nutritional product for adjunct DM therapy.

Cancer is a major global disease where abnormal cells rapidly proliferate, having the ability to migrate to different parts of the human body via a process called metastasis. Cancer is also one of the leading causes of death worldwide and is a burden financially and on the quality of human lives in both well-developed and less-developed countries, especially as the population is increasing. Until now, cancer research has focused on the search for curative treatments, and few studies have aimed at developing preventive strategies. Chemoprevention is an old concept that consists in the use of drugs, vitamins, or nutritional supplements to reduce the risk of developing or having a recurrence of cancer. The consumption of whole plant foods as chemopreventive agents is highly recommended in the dietary guidelines on the basis of health benefits from dietary phytochemicals observed in epidemiological studies. The paper entitled “Sulforaphane Modulates AQP8-Linked Redox Signaling in Leukemia Cells” of the authors C. Prata et al. (published in Vol. 2018) evaluate the potential anticancer activity of sulforaphane (SFN) in the B1647 leukemia cell line, focusing on AQP8 function and expression. The authors also investigated the effect of SFN on Nox2, Nox4, and peroxiredoxin expression and on the phosphorylation state of VEGFR-2 and Akt. X. Yang et al. (published in Vol. 2018), through another approach, investigated the antitumor activity and structural characteristics of water-soluble polysaccharides from *Kaempferia galanga* L. (KGPs) in the paper “Structural Characterization and Antitumor Activity of Polysaccharides from *Kaempferia galanga* L.”. The results showed that KGPs were acidic polysaccharides (total sugar of 85.23%, uronic acid of 24.17%) withskeletal modes of pyranose rings and mainly composed of arabinose and galactose with the average molecular weight of 8.5 × 10^5^ Da. The in vivo antitumor test showed that KGPs could effectively protect the thymus and spleen of tumor-bearing mice from solid tumors and enhance the immunoregulatory capability of CD4+ T cells and the cytotoxic effects of CD8+ T cells and NK cells, finally leading to the inhibitory effects on H22 solid tumors.

## 3. Bone Health and Polyphenols

Bone is a dynamic organ that undergoes continuous remodeling by the coordination and balance between resorption and the formation activities of osteoclast and osteoblast cells. It is well established that women are vulnerable to bone loss especially during and after menopause. The paper entitled “A Double-Blind, Placebo-Controlled Randomized Trial Evaluating the Effect of Polyphenol-Rich Herbal Congee on Bone Turnover Markers of the Perimenopausal and Menopausal Women”, by J. Wattanathorn et al. (published in Vol. 2018), tested the hypothesis that the polyphenol-rich herbal congee containing the combined extract of *Morus alba* and *Polygonum odoratum* leaves should improve bone turnover markers in menopausal women. The authors performed a randomized, double-blind, placebo-controlled study. The study demonstrates the antiosteoporotic effect of the polyphenol-rich herbal congee by a possible mechanism via the improved bone turnover via increased bone formation and decreased bone resorption.

Osteoporosis is a disease related to excessive bone resorption due to estrogen insufficiency that occurs post menopause. Primary osteoporosis, which is classified as type I (postmenopausal osteoporosis) and is frequently associated with fenestrated trabecular bone resorption, occurs between the ages of 50 and 65 years in postmenopausal women. Estrogen deficiency induces receptor activator of nuclear factor *κ*B ligand (RANKL), the key molecule required for osteoclast differentiation, leading to enhanced osteoclast activation and reduced osteoclast apoptosis. Several studies have shown that protocatechuic acid (PCA) has beneficial effects on osteoblast and osteoclast cells in vitro. S.-A. Jang et al. investigated the antiosteoporotic activity of PCA supplementation, which was determined in ovariectomized (OVX) female ICR mice at 12 weeks after OVX. In the paper “Protocatechuic Acid Attenuates Trabecular Bone Loss in Ovariectomized Mice” (published in Vol. 2018), the authors demonstrated an inhibitory potential of PCA against osteoclastogenesis, which augments bone resorption in OVX or postmenopausal conditions. This was demonstrated in the OVX mouse model. The underlying mechanism of PCA in the suppression of bone loss in OVX mice may be associated with the following effects: (1) reduction of the serum level of RANKL and increase in OPG; (2) blocking the RANK signaling pathway via downregulation of TRAF6 and NFATc1 expression; and (3) attenuation of cathepsin K and calcitonin receptor expression.

## 4. Neuroprotective Effects of Bioactive Compounds

Neuroinflammation is the key mediator of secondary brain damage in most of the neurological disorders, such as Alzheimer's disease (AD), Prion disease, Parkinson's disease (PD), multiple sclerosis (MS), ischemic stroke, experimental autoimmune encephalomyelitis (EAE), and neuropathic pain. Neuroinflammation is induced by aging-dependent conditions and aging-independent pathological events, which share similar inflammatory cascades. Ischemic cardiovascular disease (also known as “ischemic stroke”) is the third leading cause of death and disability worldwide. The number of patients suffering from cerebral ischemic disease worldwide has increased by 2 million per year, and the morbidity associated with this disease can affect young people. Therefore, searching for natural products for the protection and treatment of transient cerebral ischemia-reperfusion injury (TCI-RI) and exploring their mechanism of action are a rational approach. The paper entitled “The Protective Effect of the Total Flavonoids of *Abelmoschus esculentus* L. Flowers on Transient Cerebral Ischemia-Reperfusion Injury is Due to Activation of the Nrf2-ARE Pathway”. Therefore, Y. Luo et al. (published in Vol. 2018) examined the protective effect of an extract of the total flavonoids of *A. esculentus* flowers (AFF) on TCI-RI and its potential mechanism. The authors demonstrated that AFF had protective effects against TCI-RI possibly by direct (scavenging free radicals) and indirect (activating the neuronal Nrf2-ARE pathway to modulate damage by oxidative stress) actions.

Alzheimer's disease (AD), a progressive neurodegenerative disease, is characterized by extracellular senile plaque deposits, intracellular neurofibrillary tangles, and neuronal apoptosis. Oxidative damage is known to play an important role in neuronal damage, due to the neurodegeneration promoted by highly reactive compounds. Amongst the potential neuroprotective phytomedicines is *Caryocar brasiliense* (Camb), a Caryocaraceae family member popularly known as “pequi.” T. S. de Oliveira et al. (published in Vol. 2018) investigated the antioxidant and anticholinesterase activities as well as the neuroprotective effects of *C. brasiliense* leaf extracts, in order to provide new information on the potential use of this plant against neurodegenerative disorders in the work “Neuroprotective Effect of *Caryocar brasiliense* Camb. Leaves Is Associated with Anticholinesterase and Antioxidant Properties”. Progressive loss of memory and other cognitive functions are typical symptoms in AD. According to the amyloid hypothesis, amyloid-*β*- (A*β*-) related toxicity and imbalance are cardinal reasons that contribute to synaptic dysfunction and subsequent neurodegeneration in AD. Therefore, A*β* has been suggested as a potential therapeutic target for AD treatment. Thus, within this context the paper of the authors H. Huang et al. entitled “Procyanidins Extracted from Lotus Seedpod Ameliorate Amyloid-*β*-Induced Toxicity in Rat Pheochromocytoma Cells” (published in Vol. 2018) verify the anti-A*β* effects and protective mechanisms as a promising natural product for AD treatment. The authors evaluated the amelioration of LSPC in A*β*25-35-induced damage on rat pheochromocytoma (PC12) cells. CREB/BDNF signaling and antioxidant activity were studied as possible pathways. We used LC-MS/MS to analyze its distribution in vivo.

Microglia, neurons, astrocytes, and oligodendrocytes are the basic cells of the brain. Microglia and astrocytes, as glial cells, have a role to defend against brain injury, to maintain homeostasis, and to repair brain injury. In aging-dependent conditions and aging-independent disorders such as AD and stroke, neuroinflammation can be initiated by chronic microglial activation. Activated microglia are required for basic immune defense in the brain; however, chronic microglial activation is toxic to the central nervous system (CNS). Hence, natural compounds or nutraceuticals with the potential to regulate these steps to control microglial activation will be promising candidates for inhibiting neuroinflammation and neurodegenerative conditions. L. Subedi et al. (published in Vol. 2018) compared the efficacy of normal Dongjin rice (NR), modified resveratrol-enriched rice (RR), and resveratrol in terms of cytotoxicity and anti-inflammatory potential in activated microglia and elucidated the possible mechanisms underlying the antineuroinflammatory potential of RR in lipopolysaccharide- (LPS-) stimulated BV2 murine microglial cells. The paper “Genetically Engineered Resveratrol-Enriched Rice Inhibits Neuroinflammation in Lipopolysaccharide-Activated BV2 Microglia via Downregulating Mitogen-Activated Protein Kinase-Nuclear Factor Kappa B Signaling Pathway” not only discovered the safe and effective role of RR against aging and neuroinflammation, but also identified the antineuroinflammatory potential of NR itself because of the presence of active phytochemicals such as *α*-tocopherol and *γ*-tocopherol in rice. The anti-inflammatory effect of RR treatment seems to be mediated through the inhibition of nitrite production, MAPK phosphorylation, NF*κ*B-mediated production of proinflammatory cytokines, and expression of inflammatory proteins.

